# Metabolomics approach by ^1^H NMR spectroscopy of serum reveals progression axes for asymptomatic hyperuricemia and gout

**DOI:** 10.1186/s13075-018-1600-5

**Published:** 2018-06-05

**Authors:** Yannan Zhang, Huanzhen Zhang, Dong Chang, Fuchuan Guo, Hongzhi Pan, Yuexin Yang

**Affiliations:** 1 Department of Nutrition and Food Hygiene, School of Public Health, Ningxia Medical University, Yinchuan, 750004 China; 2 Department of Obstetrics and Gynecology, Tai’an Hospital of Traditional Chinese Medicine, Tai’an, 271000 China; 3 Department of Clinical Laboratory, Shanghai Pudong Hospital, Fudan University Pudong Medical Center, Shanghai, 201301 China; 4 Department of Nutrition and Food Safety, School of Public Health, Fujian Medical University, Fuzhou, 350122 China; 5 Department of Sanitary Inspection, Shanghai University of Medical & Health Sciences, Shanghai, 201318 China; 6 National Institute for Nutrition and Health, Chinese Center for Disease Control and Prevention, Beijing, 100050 China

**Keywords:** Metabolomics, NMR, Hyperuricemia, Gout, Biomarkers

## Abstract

**Background:**

Gout is a metabolic disease and is the most common form of inflammatory arthritis affecting men. However, the pathogenesis of gout is still uncertain, and novel biomarkers are needed for early prediction and diagnosis of gout. The aim of this study was to develop a systemic metabolic profile of patients with asymptomatic hyperuricemia (HUA) and gout by using a metabolomics approach, and find potential pathophysiological mechanisms of and markers of predisposition to gout.

**Methods:**

Serum samples were collected from 149 subjects, including 50 patients with HUA, 49 patients with gout and 50 healthy controls. ^1^H nuclear magnetic resonance (NMR) spectroscopy combined with principal components analysis and orthogonal partial least squares-discriminant analysis were used to distinguish between samples from patients and healthy controls. Clinical measurements and pathway analysis were also performed to contribute to understanding of the metabolic change.

**Results:**

By serum metabolic profiling, 21 metabolites including lipids and amino acids were significantly altered in patients with HUA or gout. The levels of identified biomarkers together with clinical data showed apparent alteration trends in patients with HUA or gout compared to healthy individuals. According to pathway analysis, three and five metabolic pathways were remarkably perturbed in patients with HUA or gout, respectively. These enriched pathways involve in lipid metabolism, carbohydrate metabolism, amino acids metabolism and energy metabolism.

**Conclusions:**

Taken together, we identified the biomarker signature for HUA and gout, which provides biochemical insights into the metabolic alteration, and identified a continuous progressive axis of development from HUA to gout.

**Electronic supplementary material:**

The online version of this article (10.1186/s13075-018-1600-5) contains supplementary material, which is available to authorized users.

## Background

Gout is a type of common inflammatory arthritis in adults that is associated with excruciating pain, and the prevalence of gout has risen over the last few decades [1–3]. It reduces quality of life in patients, even causing disability due to excruciatingly painful acute attacks of gouty arthritis, malformation of joints, chronic joint damage and renal stone formation. Gout is triggered by the deposition of monosodium urate crystals in the joints and hyperuricemia (HUA) contributes to the development of gout [4, 5]. HUA has long been recognized as the key causal precursor in the development of gout and the prevalence of comorbidities tends to increase with serum uric acid (SUA) levels [6, 7]. HUA and gout are closely associated with components of metabolic syndrome, kidney injury and cardiovascular diseases [8–10]. However, the pathogenesis of gout seems to be complex because many individuals with HUA form monosodium urate crystals and develop acute attacks of gouty arthritis, but some of them do not follow this trend [11]. It is well-known that HUA and gout are metabolic diseases, but it is uncertain what the metabolic difference between them is and whether this difference promotes acute attacks of gouty arthritis. Therefore, it is important to recognize metabolism-related indicators in gout, which may contribute to understanding of the predisposition to gout. Moreover, due to the possibility that the diagnosis of gout based on SUA as a biomedical indicator in the clinic may be inaccurate, even if crystals are identified in the digits [12], the identification of novel biomarkers associated with the occurrence and development of gout is highly desirable to prevent the acute attacks of gouty arthritis and destruction of the joints.

Metabolomics is an emerging and rapidly developing field that offers an efficient approach to describe biomarkers or characterize perturbations of diseases via detection, identification and quantification of low-molecular-weight metabolites (< 1 kDa) in biological samples such as plasma and urine [13–15]. The metabolomics approach has been successfully applied in recent years to identify early signals or biomarkers of abnormalities [16], biological pathway characterization [17] and disease diagnosis [16, 18]. ^1^H nuclear magnetic resonance (NMR) spectroscopy is an attractive tool in metabolomics research because of several advantages, such as simple sample preparation, high reproducibility and fast analysis. Therefore, ^1^H NMR-based metabolomics is suitable for simultaneous and systemic analysis of multiple compounds of metabolite fingerprinting [19].

In the current study, we carried out a serum metabolomics study on a male population with normal SUA, hyperuricaemia and gout by using ^1^H NMR spectroscopy coupled with chemometric methods. The aim of this study was to explore the serum metabolic alteration in patients with asymptomatic hyperuricemia and patients with gout, to capture the metabolic alteration associated with the initiation and progression of gout.

## Methods

### Study subjects and sample collection

Asymptomatic patients with hyperuricemia (n = 50) and patients with gout (n = 49) were enrolled in the physical examination center of the Second Affiliated Hospital of Harbin Medical University between January 2013 and June 2015. An additional 50 healthy control samples were collected from healthy donors. All the diagnosis of these patients was confirmed by experienced doctors according to serum uric acid levels and joint swelling. Hyperuricemia in men was defined as SUA ≥ 416 mmol/L, which is a widely accepted diagnostic criterion [20–23]. All patients with gout fulfilled the 1977 preliminary American Rheumatism Association classification criteria for gout [24]. They had been newly diagnosed with gout within the last 2 years and had experienced no acute attacks of gout within the last 3 months, thus excluding the effects of inflammation and drug treatments. All participants were male adult residents in the Harbin regionand were did not have diabetes mellitus, heart disease, liver or renal dysfunction, gastrointestinal disease, pulmonary disease or cancer, and had taken no metabolic drugs or dietary supplements within the last 3 months. Each participant had been given a standardized diet plan for 3 days before blood was drawn, and the consumption of alcohol and caffeine products were forbidden during this period. Demographic data (age and gender) and anthropometric data (height, weight, diastolic blood pressure (DBP) and systolic blood pressure (SBP)) were obtained from all participants, and current medications and medical history were also recorded.

Venous blood was taken from participants after overnight fasting and allowed to clot for 30 min at room temperature. It was then centrifuged at 3000 rpm for 10 min and the supernatant was stored at − 80 °C until NMR analysis.

### Clinical chemistry measurements

Alanine aminotransferase (ALT), aspartate aminotransferase (AST), total protein, albumin, globulin, fasting glucose, creatinine, urea nitrogen, triglyceride, uric acid, cholesterol, high-density lipoprotein (HDL)-cholesterol and low-density lipoprotein (LDL)-cholesterol were measured using an automatic biochemical analyzer (AUTOLAB PM4000, Rome, Italy). The values were expressed as mean ± SD. Student’s *t* test was conducted to compare the clinical biochemical data using SPSS 20 software (SPSS Inc., Chicago, IL, USA). A *p* value <0.05 was regarded as statistically significant.

### Serum sample preparation

Serum samples were removed from − 80 °C storage and thawed at 4 °C. A volume of 200 μL serum and 350 μL of 0.9% NaCl (*w*/*v*) solution containing 20% D_2_O were mixed and then followed by centrifugation (10,000 g, 4 °C, 10 min). Finally 500 μL of the supernatant of each sample was transferred into individual 5-mm high-quality NMR tubes.

### ^1^H NMR spectroscopic analysis

NMR spectra of serum samples were recorded on a Bruker AVIII 500 spectrometer (Bruker Biospin, Rheinstetten, Germany) equipped with a 5-mm inverse broadband probe at 300 K. The ^1^H NMR spectra were recorded with the relaxation edited Carr–Purcell–Meiboom–Gill (CPMG, RD-90°-(τ-180°-τ)n-acquisition) pulse sequence to detect low-molecular-weight metabolites over a spectral width of 20 ppm with 128 transients, 60 k data points, and 4 s relaxation delay. In order to facilitate the identification of metabolites, two dimensional (2D) J-resolved spectroscopy (JRES) spectra were acquired as previously reported [25, 26].

### NMR data processing

NMR spectra were processed using TOPSPIN software package (version 3.2, Bruker Biospin, Germany). For ^1^H NMR spectra, an exponential window function was employed with a line broadening factor of 0.3 Hz and zero-filled to 128 k prior to Fourier transformation. Each spectrum was then manually phase-corrected and baseline-corrected and calibrated with the anomeric proton signal of α-glucose (δ 5.23 ppm). The spectra were segmented into regions with a width of 0.01 ppm (δ 0.5–9.0 ppm) using AMIX software package (V3.9.14, Bruker Biospin). The regions of imperfect water saturation signals (δ 4.50–5.15 ppm) and urea signals (δ 5.50–6.50 ppm) were discarded. The NMR resonances were assigned according to an electronic database (HMDB, http://www.hmdb.ca/) and data from the literature [27, 28], and were confirmed with 2D NMR results.

### Multivariate statistical analysis

Multivariate data analysis was performed in order to establish a systemic overview of the discrimination of metabolic patterns in patients with HUA, patients with gout and controls. At first, principal components analysis (PCA) was used to observe the intrinsic metabolic variation in ^1^H NMR spectra data. Next, orthogonal partial least squares-discriminant analysis (OPLS-DA) was carried out to maximize the variation between groups and then detect significant metabolites that contribute to the variation. A coefficient of variation-analysis of variance (CV-ANOVA) approach was further applied to test the significance of intergroup differentiations (*p* < 0.05) in OPLS-DA models. Loadings plots of OPLS-DA models were generated using MATLAB 7.1 (Mathworks Inc., USA) with correlation coefficients. In these loadings plots, hot-colored metabolites have greater contribution in intergroup differentiations than cold-colored ones. The selection of significant metabolites was based on correlation coefficients (|r| > 0.6) and Student’s *t* test (*p* < 0.01). To visualize the alterations of remarkable metabolites in three groups, a heat map was created using MetaboAnalyst 3.0 (http://www.metaboanalyst.ca/).

### Pathway analysis

Pathway analysis of remarkably changed metabolites in patients with HUA and patients with gout was applied within MetaboAnalyst 3.0. Among all the perturbed pathways, the ones with impact value > 0.1 and *p* < 0.05 were selected as significantly perturbed metabolic pathways in HUA and patients with gout.

## Results

### Baseline characteristics of participants

The representative sample of this metabolomics study consisted of 149 male participants (50 controls, 50 patients with HUA and 49 patients with gout) whose basic characteristics and clinical variables are summarized in Table 1. Body mass index (BMI), DBP, SBP, ALT, AST, fasting glucose, uric acid, triglyceride, cholesterol and LDL-cholesterol were notably increased in HUA and in patients with gout compared to the control group (*p* < 0.05). Compared to the HUA group, the gout group had significant higher levels of DBP, SBP, fasting glucose, uric acid and HDL-cholesterol and a lower level of albumin.

**Table 1:** Baseline characteristics (demographic, anthropometric and clinical data) for the HUA, gout, and control groups

Parameter	Control (n = 50)	HUA (n = 50)	Gout (n = 49)
Basic characteristics			
Age (years)	43.8 ± 11.5	39.08 ± 10.4^*^	45.6 ± 7.3^##^
Sex (female/male)	0/50	0/50	0/50
BMI (kg/m^2^)	23.4 ± 3.2	27.09 ± 3.0^**^	26.5 ± 3.3^**^
Smoker/non-smoker	24/26	22/28	23/26
Alcohol consumption (%)	61	67	57
Clinical variables			
DBP (mmHg)	78.6 ± 5.7	85.1 ± 11.1^**^	91.8 ± 12.4^**##^
SBP (mmHg)	114.8 ± 6.5	125.8 ± 14.4^**^	136.3 ± 19.2^**##^
ALT(U/L)	22.6 ± 11.3	43.8 ± 30.6^**^	34.8 ± 18.9^**^
AST(U/L)	20.5 ± 5.9	27.7 ± 14.8^**^	26.8 ± 12.9^**^
Total protein (g/L)	74.8 ± 4.3	75.5 ± 4.4	74.6 ± 4.5
Albumin (g/L)	49.4 ± 2.9	48.9 ± 2.2	47.8 ± 3.3^**#^
Globulin (g/L)	25.4 ± 4.0	26.7 ± 3.5	26.9 ± 4.0
Fasting glucose (mmol/L)	5.0 ± 0.4	5.7 ± 0.7^**^	6.3 ± 1.9^**#^
Urea nitrogen (mmol/L)	5.6 ± 1.3	5.4 ± 1.2	5.5 ± 1.8
Creatinine (μmol/L)	80.6 ± 9.6	81.2 ± 13.9	91.8 ± 23.2^**##^
Uric acid (μmol/L)	325.1 ± 60.6	470.6 ± 55.0^**^	536.2 ± 131.4^**##^
Triglyceride (mmol/L)	1.1 ± 0.4	2.3 ± 1.2^**^	3.1 ± 2.1^**^
Cholesterol (mmol/L)	4.7 ± 0.8	5.2 ± 0.8^**^	5.4 ± 1.0^**^
HDL-cholesterol (mmol/L)	1.4 ± 0.3	1.1 ± 0.2^**^	1.3 ± 0.3^##^
LDL-cholesterol (mmol/L)	2.7 ± 0.5	3.2 ± 0.7^**^	3.0 ± 0.6^*^

Data are presented as mean ± SD except where stated otherwise

*HUA* hyperuricemia, *BMI* body mass index, *DBP* diastolic blood pressure, *SBP* systolic blood pressure, *ALT* alanine aminotransferase, *AST* aspartate aminotransferase, *HDL* high-density lipoprotein, *LDL* low-density lipoprotein

^*^p < 0.05, ^**^p < 0.01, compared to control; ^#^*p* < 0.05, ^##^*p* < 0.01, compared to the HUA group

### ^1^H NMR spectroscopy

**Fig. 1:**
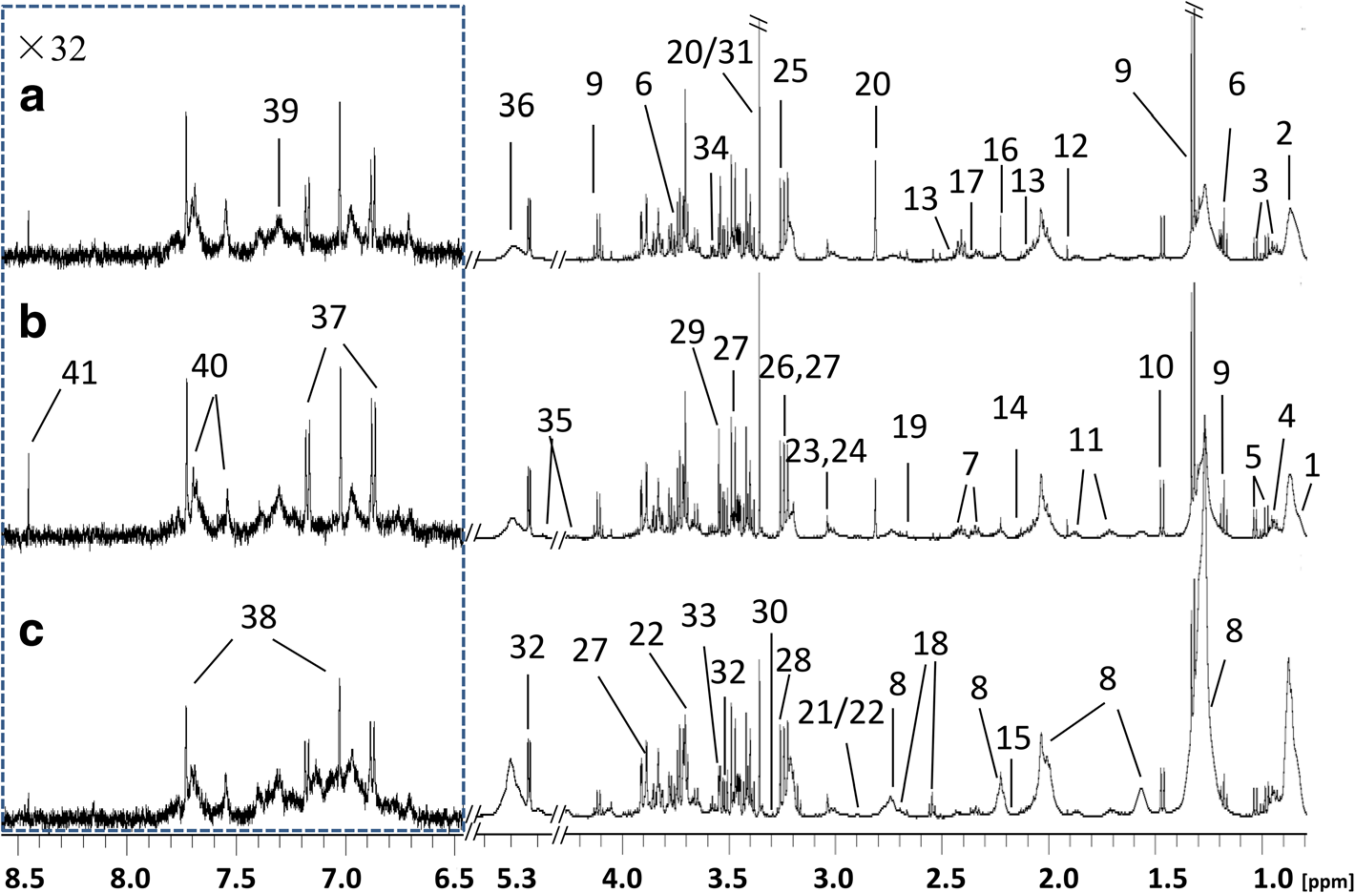
Typical 500-MHz Carr–Purcell–Meiboom–Gill (CPMG) ^1^H nuclear magnetic resonance spectra of human serum samples from controls (**a**), patients with hyperuricemia (**b**) and patients with gout (**c**). The dotted regions were vertically expanded 32 times. 1, high-density lipoprotien; 2, very low-density lipoprotein; 3, isoleucine; 4, leucine; 5, valine; 6, ethanol; 7, 3-hydroxybutytrate; 8, lipid; 9, lactate; 10, alanine; 11, lysine; 12, acetate; 13, glutamine; 14, methionine; 15, glycoprotein; 16, acetone; 17, glutamate; 18, citrate; 19, aspartate; 20, methylguanidine; 21, trimethylamine; 22, dimethylglycine; 23, creatine; 24, creatinine; 25, choline; 26, arginine; 27, β-glucose; 28, trimethylamine n-oxide; 29, myo-inositol; 30, proline; 31, scyllo-inositol; 32, α-glucose; 33, glycine; 34, threonine; 35, triglycerides; 36, unsaturated lipids; 37, tyrosine; 38, 1-methylhistidine; 39, phenylalanine; 40, tryptophan; 41, formate

Three CPMG ^1^H NMR spectra (Fig. 1) of serum samples obtained from control individuals (Fig. 1a), patients with HUA (Fig. 1b) and gout (Fig. 1c) show the average signals of metabolites. In total, 41 metabolites were identified in serum samples including lipids, glucose, amino acids and organic acids, as shown in Fig. 1 and Additional file 1.

### Multivariate analysis of NMR data

Since SUA is an important factor in gout, and the range of SUA levels was large in subjects with gout in this study (Table 1), the NMR spectrum data from the two gout subgroups including gout with HUA (n = 32) and gout with normal SUA (n = 17) were analyzed by PCA and OPLS-DA, to determine whether SUA affected the metabolic profiles in patients with gout. The OPLS-DA scores plot (Additional file 2) showed no metabolic variation trend between the two subgroups in relation to SUA. This may be due to the complexity of gout pathogenesis, which cannot be explained by levels of uric acid, as aforementioned. Therefore, in the following multivariate data analysis, the two gout subgroups were processed and treated as one group.

**Fig. 2:**
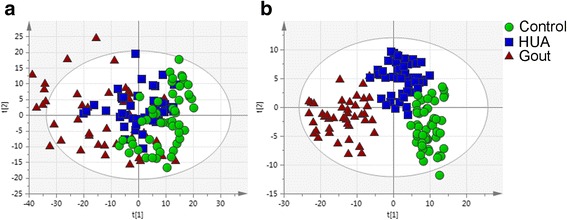
Principal components analysis (**a**) and orthogonal partial least squares-discriminant analysis (**b**) score plots based on ^1^H nuclear magnetic resonance data from serum samples obtained from controls, patients with hyperuricemia (HUA) and patients with gout

To observe the clustering trends of samples obtained from patients with HUA or gout and control subjects, serum metabolic profiling was performed using PCA and OPLS-DA. The PCA scores plot for the first two components (*R*^2^X = 0.367, *Q*^2^ = 0.34) reflecting a separation trend in the gout, HUA and control groups (Fig. 2a). Furthermore, three distinct clusters of samples were observed in the OPLS-DA scores plot (*R*^2^X = 0.484, *R*^2^Y = 0.711 and *Q*^2^ = 0.566; Fig. 2b). To assess the risk that the current OPLS-DA model was spurious, the data were analyzed using the CV-ANOVA approach; a *p* value of 1.1E-26 showed that the OPLS-DA model was valid.

**Fig. 3:**
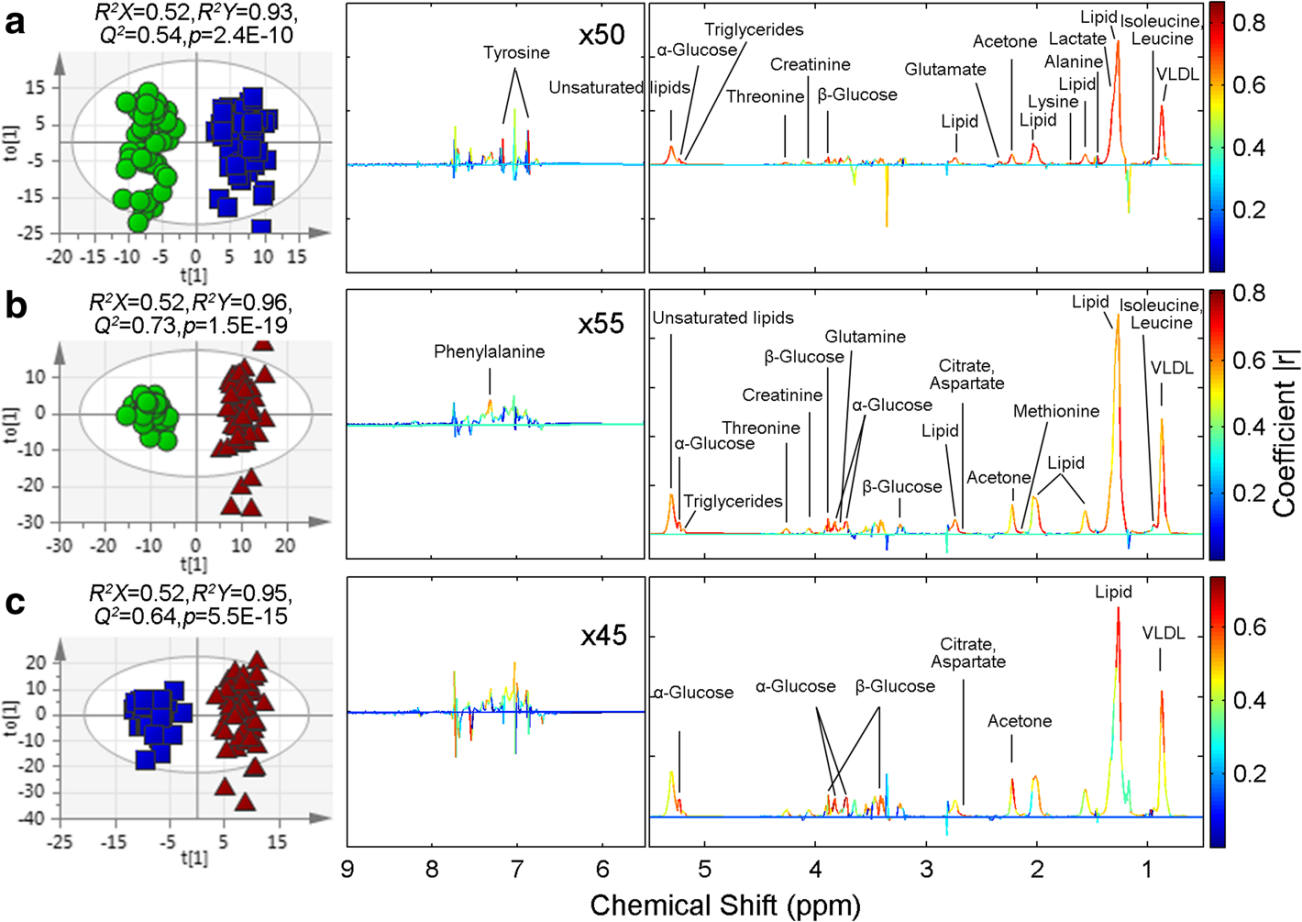
Orthogonal partial least squares-discriminant analysis score plots of samples (left panel) and corresponding coefficient loading plots (right panel) obtained from different pairwise groups: **a** hyperuricemia (HUA) (blue dots) and control groups (green dots); **b** gout (red dots) and control groups (green dots); **c** gout (red dots) and HUA (blue dots). The color bar on the right corresponds to the weight of a variable in the discrimination between sets of samples, beginning from weak (blue) to strong (red) correlation for the discrimination. VLDL, very low-density lipoprotein

**Fig. 4:**
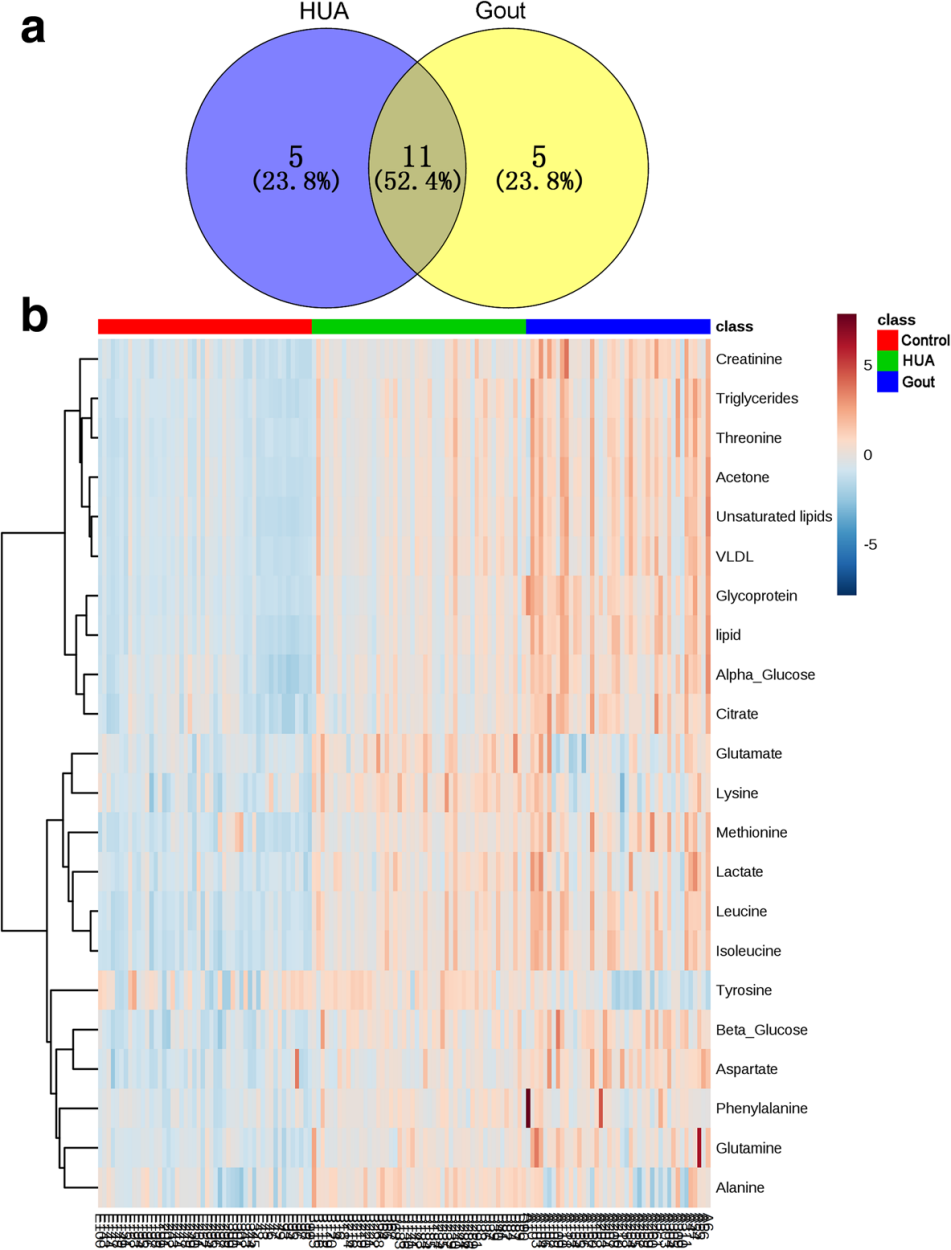
Significantly changed metabolites in patients with hyperuricemia (HUA) and patients with gout. **a** Numbers of significant metabolites. **b** Heatmap of significantly changed metabolites. The color of each section corresponds to a concentration value of each metabolite calculated by the peak area normalization method (red, upregulated; blue, downregulated)

OPLS-DA was further performed to identify the significantly altered metabolites in the HUA and gout groups as shown in Fig. 3. The OPLS-DA scores plot (*R*^2^X = 0.516, *Q*^2^Y = 0.931, *Q*^2^ = 0.538, *p* = 2.4E-10) showed clear separation between the HUA and control groups (Fig. 3a). According to the corresponding loading plot, compared with control group, the HUA group had significantly higher levels of very low-density lipoprotein (VLDL), isoleucine, leucine, lipid, lactate, alanine, lysine, acetone, glutamate, creatinine, β-glucose, α-glucose, threonine, triglycerides, unsaturated lipids and tyrosine. The metabolic differences between the gout and control groups were visible in the OPLS-DA scores plot (*R*^2^X = 0.52, *R*^2^Y = 0.963, *Q*^2^ = 0.729, *p* = 1.5E-19; Fig. 3b). Compared with the control group, VLDL, isoleucine, leucine, lipid, glutamine, methionine, acetone, citrate, aspartate, β-glucose, creatinine, α-glucose, threonine, triglycerides, unsaturated lipids and phenylalanine were remarkably increased in the gout group. Moreover, there was a clear difference in metabolic profiles between the HUA and gout groups in the OPLS-DA scores plot (*R*^2^X = 0.518, *R*^2^Y = 0.945, *Q*^2^ = 0.641, *p* = 5.5E-15; Fig. 3c). Compared with the HUA group, the gout group had notably higher VLDL, lipid, acetone, citrate, aspartate, β-glucose and α-glucose. The significantly changed metabolites are summarized in Table 2. Among the 21 metabolites remarkably changed in patients with HUA and patients with gout, a total of 11 metabolites were disturbed in both groups (Fig. 4a). To further understand the metabolic changes in patients with HUA and patients with gout, a clustering heatmap was used to visualize changes in metabolites. The heatmap (Fig. 4b) of 21 significantly changed metabolites in patients with HUA and patients with gout, showed that there was a remarkable change of the metabolic profile in patients with HUA and a more greater difference in patients with gout.

**Table 2:** Summary of significantly changed metabolites in the HUA and gout group

Metabolites	Changes in HUA (vs control)			Changes in gout (vs control)			Changes in gout (vs HUA)		
	Trend	*r* ^*a*^	*p* ^*b*^	Trend	*r* ^*a*^	*p* ^*b*^	Trend	*r* ^*a*^	*p* ^*b*^
VLDL	↑	0.71	0.00	↑	0.61	0.00	↑	0.61	0.00
Isoleucine	↑	0.86	0.00	↑	0.72	0.00	–	–	–
Leucine	↑	0.83	0.00	↑	0.71	0.00	–	–	–
Lipid	↑	0.68	0.00	↑	0.72	0.00	↑	0.63	0.00
Lactate	↑	0.70	0.00	–	–	–	–	–	–
Alanine	↑	0.77	0.00	–	–	–	–	–	–
Lysine	↑	0.76	0.00	–	–	–	–	–	–
Glutamine	–	–	–	↑	0.60	0.00	–	–	–
Methionine	–	–	–	↑	0.67	0.00	–	–	–
Acetone	↑	0.64	0.00	↑	0.63	0.00	↑	0.65	0.00
Glutamate	↑	0.69	0.00	–	–	–	–	–	–
Citrate	–	–	–	↑	0.68	0.00	↑	0.65	0.00
Aspartate	–	–	–	↑	0.73	0.00	↑	0.63	0.00
Creatinine	↑	0.63	0.00	↑	0.65	0.00	–	–	–
β-Glucose	↑	0.65	0.00	↑	0.70	0.00	↑	0.65	0.00
α-Glucose	↑	0.63	0.00	↑	0.68	0.00	↑	0.66	0.00
Threonine	↑	0.65	0.00	↑	0.60	0.00	–	–	–
Triglycerides	↑	0.70	0.00	↑	0.61	0.00	–	–	–
Unsaturated lipids	↑	0.67	0.00	↑	0.63	0.00	–	–	–
Tyrosine	↑	0.75	0.00	–	–	–	–	–	–
Phenylalanine	–	–	–	↑	0.63	0.00	–	–	–

Increased levels are indicated by arrows (↑)

*VLDL* very low-density lipoprotein

^a^Correlation coefficient (*r*) was obtained from the orthogonal partial least squares-discriminant analysis model

^b^The *p* value was calculated using Student’s *t* test

### Pathway analysis

**Fig. 5:**
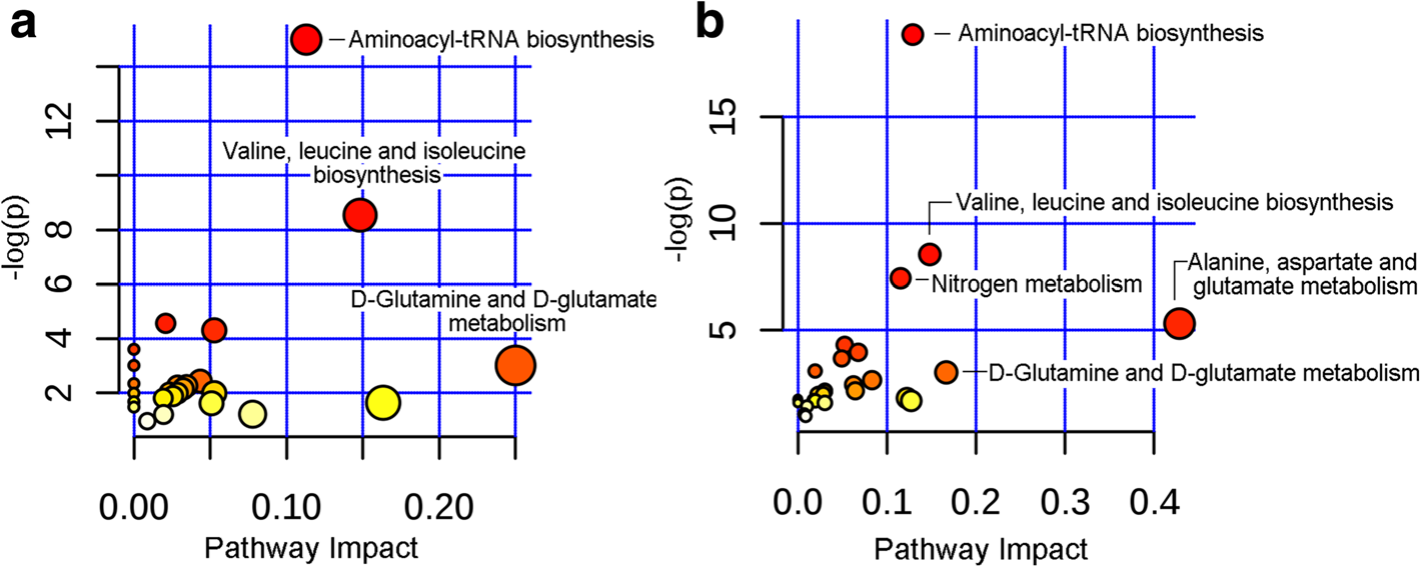
Pathway analysis of significantly changed metabolites in the hyperuricemia group (**a**) and gout group (**b**). tRNA, transfer RNA

According to the pathway analysis, 29 and 30 metabolic pathways were disturbed in patients with HUA and patients with gout, respectively. In the HUA group, three pathways were significantly perturbed including aminoacyl-transfer RNA (tRNA) biosynthesis, valine, leucine and isoleucine biosynthesis, and D-glutamine and D-glutamate metabolism were significantly perturbed. In patients with gout, five metabolic pathways were remarkably disturbed including aminoacyl-tRNA biosynthesis, valine, leucine and isoleucine biosynthesis, nitrogen metabolism, alanine, aspartate and glutamate metabolism, D-glutamine and D-glutamate metabolism (Fig. 5 and Additional file 3).

## Discussion

Gout is a worldwide public health problem. However, current research falls short in evaluating the metabolic change in gout and asymptomatic hyperuricemia. In the current study, we used ^1^H NMR-based metabolomics to analyze metabolites in serum from patients with asymptomatic hyperuricemia and gout, hoping to help gain understanding of the predisposition to gout.

Our research demonstrated that the ^1^H NMR-based metabolomics approach is feasible to examine metabolic change in patients with asymptomatic hyperuricemia and gout. Such an approach is also helpful in selecting metabolic pathways that play vital roles in the development of gout. The levels of some identified biomarkers showed a trend of an apparent increase in patients with HUA and patients with gout from levels in healthy individuals; together with clinical data this suggests an increase in metabolic disorders. In pathway analysis (Fig. 5 and Additional file 3), more metabolic pathways were notably affected in the gout groups (five pathways) than in the HUA groups (three pathways), which indicated that there was a more severe metabolic disorder in patients with gout.

### Lipid metabolism

Altered lipid profiles were observed in patients with HUA, including increased levels of VLDL, fatty acids, triglyceride (TG) and unsaturated lipids; related variables in clinical chemistry results (Table 1) were also found to be significantly changed including increased TG, total cholesterol (TC), LDL-cholesterol and decreased HDL-cholesterol. Similarly, elevated VLDL, fatty acids, TG and unsaturated lipids were observed in patients with gout, which was consistent with clinical chemistry data showing that TC, TG and LDL-cholesterol increased in patients with gout. These results suggest that there was a lipid metabolism disorder in both patients with HUA and patients with gout. Several researchers have reported that HUA and gout are associated with cardiovascular and cerebrovascular diseases due to the correlation between serum uric acid and serum lipids [29, 30]. Moreover, our findings that lipid metabolism disorder and the elevated blood pressure, fasting plasma glucose and BMI in patients with HUA and patients with gout were consistent with the previous study showing that the prevalence of metabolic syndrome among individuals with HUA and gout is remarkably high [29, 31]. Our results indicate that lipid levels are highly linked with HUA and gout, thereby lipid-lowering therapy may provide a supplementary role to slow the development of gout.

### Carbohydrate metabolism

Increased α-glucose and β-glucose were observed in samples from both patients with HUA and patients with gout, which suggests changes in carbohydrate metabolism. A large number of studies have shown that serum uric acid is positively related to elevated blood glucose due to insulin resistance [32–35]. Insulin is the only hormone in the body that promotes the uptake and utilization of glucose in tissues and lowers blood glucose. Although we did not measure insulin, the increased glucose verified the inhibition of glucose metabolism in both patients with HUA and patients with gout, and it was more severe in patients with gout because of the higher glucose in the samples from patients with gout than in those from patients with HUA. As the main product of glycolysis, lactate is typically interpreted as a marker of anaerobic metabolism, and its accumulation usually accounts for a high energy demand in the biological system [36]. Increased lactate was observed in HUA samples, indicating the energy demand in patients with HUA induced by low utilization of glucose. However, the trend of increased lactate was not observed in gout; this may be due to the accelerated gluconeogenesis in patients with gout for converting lactate to glucose to meet the more urgent energy demand. Increased citrate levels were seen in gout but not in HUA samples. Since citrate is an important intermediate in the tricarboxylic acid cycle (TCA) in mitochondria, the data may imply that altered mitochondrial function affected citrate handling and induced and imbalance in the global energy supply in patients with gout.

### Aminoacyl-tRNA biosynthesis

Six and seven amino acids were significantly increased (*p* < 0.01) in patients the HUA (alanine, lysine, isoleucine, leucine, threonine and tyrosine) and the gout (phenylalanine, glutamine, aspartic acid, methionine, isoleucine, leucine, threonine) groups, respectively (Additional file 3), which indicates decreased protein syntheses or increased amino acid synthesis. Coincidentally, aminoacyl-tRNA biosynthesis was significantly affected in both patients with gout and patients with HUA. Aminoacyl-tRNA biosynthesis plays an important role in matching amino acids with tRNAs containing the corresponding anticodon for the messenger RNA (mRNA)-guided synthesis of proteins at the ribosome [37]. As we all know, amino acid metabolism is the biochemical basis in the regulation of both proteins and energy metabolisms. Greater involvement of amino acids and the greater impact value of aminoacyl-tRNA biosynthesis in gout compared to HUA suggests that translation was suppressed following the development of gout. Furthermore, aminoacyl-tRNA synthetases (AARSs) are essential enzymes in aminoacyl-tRNA biosynthesis, which have a family of twenty enzymes [38]. It is reported that mutations in AARSs have been identified in diverse human diseases, such as musculoskeletal, cardiovascular, and urinary diseases [39]. Therefore, AARSs maybe potential indicators for identifying HUA and gout.

### Valine, leucine and isoleucine biosynthesis

Branched chain amino acids (BCAAs), including isoleucine, leucine and valine, are essential amino acids and act as important signaling molecules and substrate in protein synthesis. On the other hand, increasing evidence shows that perturbed amino acid metabolism, especially circulating metabolites such as high levels of blood BCAAs, are strongly associated with insulin resistance, obesity, diabetes mellitus and cardiovascular disease [40–42]. Mitochondrial branched chain aminotransferase (BCATm), one of the two BCAT isoforms and highly expressed in all tissues in the mitochondria of the cell, converts the BCAAs into their corresponding α-keto acids. Thus, the increased levels of BCAAs in our study can be attributed to reduced expression of BCATm. It indicates that the increase in BCAAs causes the accumulation of its byproducts that can impaire mitochondrial capacity, and the affected mitochondrial function is related to the development of insulin resistance [40, 43]. Wang et al. found that BCAAs are significantly related to obesity and risk factors for some metabolic diseases [44]. Another study followed 2422 normoglycemic individuals for 12 years and found that the BCAAs may presage the development of type 2 diabetes mellitus by up to a decade or more and thus, may be among the earliest detectable metabolic derangements on the route to diabetes mellitus [45]. Our findings are in agreement, as both the HUA and gout groups had higher levels of isoleucine and leucine, and elevated BMI and fasting glucose, suggesting that there is correlation between insulin resistance and gout development.

Furthermore, it is known that BCAAs can undergo transamination to generate nitrogen for synthesis of non-essential amino acids such as glutamine and alanine [46]. In our study, although BCAAs were increased in samples both from patients with HUA and patients with gout, increased glutamine was only observed in gout but not in HUA and increased alanine was only seen in HUA but not in gout. These may have resulted from differential consumption of these amino acids in HUA and in gout. Glutamine is the most abundant free amino acid in human blood; it is consumed by proliferating cells and converted to glutamate en route to producing other metabolic intermediates that contribute to cell growth [47, 48]. Therefore, the increased level of glutamine in gout may due to the lower cellular metabolic rate in patients with gout. Alanine is used in protein synthesis and as precursor for gluconeogenesis in the liver. Under periods of starvation, alanine is generated from muscle BCAAs and transported to the liver where it is used in the glucose alanine cycle to make glucose for energy needs [49]. Hence, the fact that increased alanine was only seen in HUA but not in gout may be due to its consumption in gluconeogenesis to meet the more urgent energy demand in patients with gout.

### D-Glutamine and D-glutamate metabolism

The D-glutamine and D-glutamate metabolism is a major regulatory mechanism of glutamate and glutamine levels in organisms [50]. Glutamate is an excitatory neurotransmitter and glutamine is the precursor and storage form of glutamate. In this study, compared to controls, patients with HUA had a higher level of glutamate and patients with gout had a higher level of glutamine. Thus, our results indicate that the perturbation of D-glutamine and D-glutamate metabolism occurred in both patients with HUA and patients with gout. There is evidence to suggest that uric acid has a remarkable antioxidant effect on neurons [51, 52]. However, the protective effect of gout on the risk of neurological disease is a controversial issue [53, 54]. It is said that metabolic syndrome, a frequent comorbidity of HUA and gout, might offset the anti-oxidative benefit from the high uric acid level [55, 56]. Zheng et al. identified disturbance of the glutamate-glutamine cycle with an increased level of glutamine in the hippocampus of mice with diabetes-associated decline in cognition, and regarded this change as the underlying reason for diabetes-related neurological complications [57]. Although none of the patients with gout in the present study had diabetes mellitus, fasting glucose was significantly increased in these patients. Thus, glutamine may be an early biomarker of gout and its comorbidities.

### Alanine, aspartate and glutamate metabolism and nitrogen metabolism

In our study, metabolites related to alanine, aspartate and glutamate metabolism (aspartic acid and glutamine) and nitrogen metabolism (phenylalanine, aspartic acid and glutamine) were increased in serum from patients with gout: this indicates the perturbation of amino acid metabolism and energy metabolism in patients with gout. Among all the significantly disturbed metabolic pathways, alanine, aspartate and glutamate metabolism and nitrogen metabolism were disturbed in patients with gout but were not detected in patients with HUA, which showed the aggravation of metabolic disorders in patients with gout.

## Conclusion

In summary, we investigated the application of ^1^H NMR spectroscopy-based metabolomics to detect metabolic changes in serum from patients with HUA and patients with gout. Our results indicated significant dysregulation of metabolic pathways in patients with gout. The metabolic alterations were associated with the disturbance of lipid metabolism, carbohydrate metabolism, amino acids metabolism and energy metabolism. Clear metabolic differences were observed between patients with HUA, patients with gout and controls, indicating that the disease has a continuous progressive development axis. The combination of these metabolic alterations may corporately hold promise for early prediction and diagnosis of the progression of gout.

## Additional files

Additional file 1: Metabolite assignments of major resonances detected in ^1^H NMR spectra from human serum samples. (DOCX 29 kb)

Additional file 2: PCA and OPLS-DA scores plots based on ^1^H NMR spectrum data of serum samples obtained from two gout subgroups including gout with HUA (n = 32) and gout with normal SUA (n = 17). (PDF 337 kb)

Additional file 3: Metabolic pathways significantly altered in patients with HUA and patients with gout. (DOCX 18 kb)

## Abbreviations

AARSs: Aminoacyl-tRNA synthetases
ALT: Alanine aminotransferase
AST: Aspartate aminotransferase
BCAAs: Branched chain amino acids
BCATm: Mitochondrial branched chain aminotransferase
BMI: Body mass index
CPMG: Carr–Purcell–Meiboom–Gill
DBP: Diastolic blood pressure
HDL: High-density lipoprotein
HUA: Asymptomatic hyperuricemia
JRES: J-resolved spectroscopy
LDL: Low-density lipoprotein
NMR: Nuclear magnetic resonance
OPLS-DA: Orthogonal partial least squares-discriminant analysis
PCA: Principal components analysis
SBP: Systolic blood pressure
SUA: Serum uric acid
TC: Total cholesterol
tRNA: Transfer RNA
TG: Triglyceride
VLDL: Very low-density lipoprotein
