# Supplementary material for: Metabolomics approach by 1H NMR spectroscopy of serum reveals progression axes for asymptomatic hyperuricemia and gout
Source: Arthritis Res Ther. 2018 Jun 5;20:111. doi: 10.1186/s13075-018-1600-5 (PMC5989453; doi:10.1186/s13075-018-1600-5)
Supplement: Supplementary file 1 — Metabolite assignments of major resonances detected in 1H NMR spectra from human serum samples. (DOCX 29 kb) [file 13075_2018_1600_MOESM1_ESM.docx]

Metabolite assignments of major resonances detected in ^1^H NMR spectra from human serum samples.

| No. | Metabolites | Chemical shift (ppm)(Multiplicity) | Assignment |
| --- | --- | --- | --- |
| 1 | HDL | 0.84 (t) | CH_3_(CH_2_)_n_ |
| 2 | VLDL | 0.88 (t) | CH_3_CH_2_CH_2_C= |
| 3 | Isoleucine | 0.93(t), 1.00(d) | δCH_3_, γCH_3_, |
| 4 | Leucine | 0.95(d), 0.96(d) | δCH_3_, δCH_3_ |
| 5 | Valine | 0.98(d), 1.03(d) | γCH_3_, γCH_3_ |
| 6 | Ethanol | 1.18(t), 3.65(q) | CH_3_, CH_2_, |
| 7 | 3-hydroxybutytrate | 1.19(d), 2.30(m), 2.40(m), 4.17(m) | γCH_3_, αCH, αCH, βCH_2_ |
| 8 | Lipid | 1.25(m), 1.57(m), 2.01(m), 2.24(m), 2.74(m) | CH_3_(CH_2_)n, CH_2_CH_2_CO, CH_2_-C=C, CH_2_-C=O, =C-CH_2_-C= |
| 9 | Lactate | 1.32(d), 4.11(q) | βCH_3_, αCH |
| 10 | Alanine | 1.46(d), 3.76(q) | βCH_3_, αCH |
| 11 | Lysine | 1.70(m), 1.88(m), 3.03(t) | γCH_2_, βCH, εCH_2_ |
| 12 | Acetate | 1.91(s) | βCH_3_ |
| 13 | Glutamine | 2.11(m), 2.45(m), 3.77(t) | βCH_2_, γCH_2_, αCH |
| 14 | Methionine | 2.13(s), 2.63(t) | δCH_3_, γCH_2_, |
| 15 | Glycoprotein | 2.18(s) | CH_3_-C=O |
| 16 | Acetone | 2.22(s) | CH_3_ |
| 17 | Glutamate | 2.35( m) | half γ-CH_2_ |
| 18 | Citrate | 2.52(d), 2.68(d) | Half CH_2_, half CH_2_ |
| 19 | Aspartate | 2.66(dd), 2.80(dd) | half β-CH_2_, half β-CH_2_ |
| 20 | Methylguanidine | 2.81(s), 3.35(s) | CH_3_, CH_3_ |
| 21 | TMA | 2.90(s) | CH_3_ |
| 22 | Dimethylglycine | 2.90(s), 3.71(s) | N-CH_3_, CH_2_ |
| 23 | Creatine | 3.03(s), 3.93(s) | N-CH_3_, CH_2_ |
| 24 | Creatinine | 3.04(s), 4.05(s) | CH_3_, CH_2_ |
| 25 | Choline | 3.22(s) | N(CH_3_)_3_ |
| 26 | Arginine | 3.24(t) | δCH_2_ |
| 27 | β-Glucose | 3.24(dd), 3.40(t), 3.46(m), 3.89(d), 4.64(d) | 2-CH, 4-CH, 5-CH, 6-CH, 1-CH |
| 28 | TMAO | 3.26(s) | CH_3_ |
| 29 | Myo-inositol | 3.27(t), 3.53(dd), 3.62(t), 4.05(t) | 2-CH, 4,6-CH, 1,3-CH, 5-CH |
| 30 | Proline | 3.33(m) | half δ-CH_2_ |
| 31 | Scyllo-inositol | 3.35(s) | CHOH |
| 32 | α-Glucose | 3.52(d), 3.71(t), 3.82(m), 5.23(d) | 2-CH, 3-CH, 5,6-CH, 1-CH |
| 33 | Glycine | 3.55(s) | CH_2_ |
| 34 | Threonine | 3.58(d), 4.26(m) | αCH, βCH_2_ |
| 35 | Triglycerides | 4.06(m), 4.27(m), 5.20(m) | CH_2_O, CH_2_’O, CHO |
| 36 | Unsaturated lipids | 5.30 (m) | CH=CH |
| 37 | Tyrosine | 6.87(d), 7.17(d) | CH, CH |
| 38 | 1-Methylhistidine | 7.03(s), 7.73(s) | 4-CH, 2-CH |
| 39 | Phenylalanine | 7.31(m), 7.35(m), 7.40(m) | 2,6-CH, 3,5-CH, 4-CH |
| 40 | Tryptophan | 7.20(t), 7.28(t), 7.53(d), 7.73(d) | 5-CH, 6-CH, 7-CH, 4-CH |
| 41 | Formate | 8.45(s) | CH |

Abbreviations: s, singlet; d, doublet; dd, double doublet; t, triplet; q, quartet; m, multiplet; HDL, high density lipoprotein; VLDL, very low density lipoprotein; TMA, Trimethylamine; TMAO, trimethylamine N-oxide.
