# Supplementary figures and images for: Metabolomics approach by 1H NMR spectroscopy of serum reveals progression axes for asymptomatic hyperuricemia and gout
Source: Arthritis Res Ther. 2018 Jun 5;20:111. doi: 10.1186/s13075-018-1600-5 (PMC5989453; doi:10.1186/s13075-018-1600-5)

A

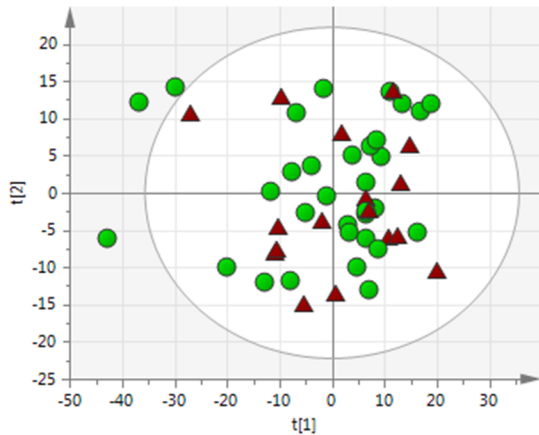

B

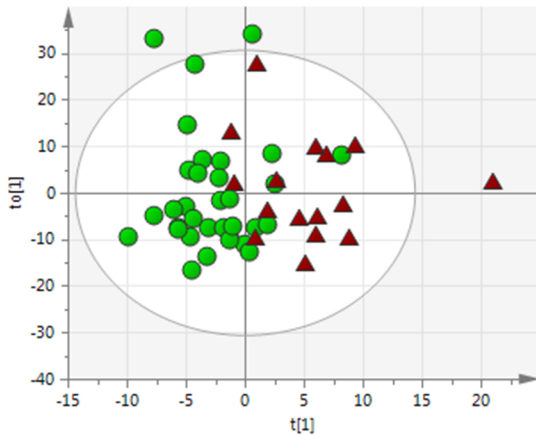

● Gout & HUA+  
▲ Gout & HUA-

Supplement: Supplementary file 2 — PCA and OPLS-DA scores plots based on 1H NMR spectrum data of serum samples obtained from two gout subgroups including gout with HUA (n = 32) and gout with normal SUA (n = 17). (PDF 337 kb) [file 13075_2018_1600_MOESM2_ESM.pdf]
