# Supplementary material for: Metabolomics approach by 1H NMR spectroscopy of serum reveals progression axes for asymptomatic hyperuricemia and gout
Source: Arthritis Res Ther. 2018 Jun 5;20:111. doi: 10.1186/s13075-018-1600-5 (PMC5989453; doi:10.1186/s13075-018-1600-5)
Supplement: Supplementary file 3 — Metabolic pathways significantly altered in patients with HUA and patients with gout. (DOCX 18 kb) [file 13075_2018_1600_MOESM3_ESM.docx]

Metabolic pathways significantly altered in HUA and gout patients.

| No. | Pathway Name | Match Status | *p* | -log(*p*) | Holm *p* | FDR | Impact |
| --- | --- | --- | --- | --- | --- | --- | --- |
|  | HUA |  |  |  |  |  |  |
| 1 | Aminoacyl-tRNA biosynthesis | 6/75 | 3.059E-7 | 15.0 | 2.448E-5 | 2.447E-5 | 0.11291 |
| 2 | Valine, leucine and isoleucine biosynthesis | 3/27 | 1.958E-4 | 8.5385 | 0.01547 | 0.007814 | 0.14815 |
| 3 | D-Glutamine and D-glutamate metabolism | 1/11 | 0.04924 | 3.0111 | 1.0 | 0.5627 | 0.25 |
|  | Gout |  |  |  |  |  |  |
| 1 | Aminoacyl-tRNA biosynthesis | 7/75 | 6.435E-9 | 18.862 | 5.148E-7 | 5.147E-7 | 0.12904 |
| 2 | Valine, leucine and isoleucine biosynthesis | 3/27 | 1.958E-4 | 8.5385 | 0.01547 | 0.007831 | 0.14815 |
| 3 | Nitrogen metabolism | 3/39 | 5.936E-4 | 7.4294 | 0.04629 | 0.01583 | 0.11538 |
| 4 | Alanine, aspartate and glutamate metabolism | 2/24 | 0.00496 | 5.3059 | 0.3821 | 0.09924 | 0.42857 |
| 5 | D-Glutamine and D-glutamate metabolism | 1/11 | 0.04924 | 3.0111 | 1.0 | 0.4377 | 0.16667 |

Note: *p* is the original *p* value calculated from the enrichment analysis. The Holm *p* is the value adjusted by Holm-Bonferroni method, FDR is the value adjusted using False Discovery Rate. The impact is the pathway impact value calculated from pathway topology analysis.
